# Comparative evaluation of GeneXpert and reverse transcription polymerase chain reaction for SARS-CoV-2 diagnosis: a retrospective study

**DOI:** 10.1099/acmi.0.000643.v5

**Published:** 2025-08-21

**Authors:** Ashish William, Yogita Rai, Deepti Rawat, Sanjib Gogoi, Megh Singh Dhakad, Manoj Jais, Ravinder Kaur

**Affiliations:** 1 Department of Microbiology, Lady Hardinge Medical College & Associated Hospitals, New Delhi-110001, Delhi, India

**Keywords:** Cq value, GeneXpert, real-time reverse transcription PCR, SARS-COV-2

## Abstract

**Background.** The novel coronavirus disease 2019 (COVID-19) has highlighted vulnerabilities in healthcare systems and has brought the world to a standstill single-handedly. Diagnostic testing for COVID-19 is critical for understanding epidemiology, contact tracing, case management and controlling the transmission of the severe acute respiratory syndrome coronavirus 2 (SARS-CoV-2). Real-time reverse transcription PCR test, the gold standard test, involves fairly complex steps and takes nearly 24–48 h to generate the results. GeneXpert is a rapid nucleic-acid-detection-based test approved by the Indian Council of Medical Research, which can shorten the turnaround time significantly.

**Aim.** The study aimed to compare the performance of GeneXpert against the gold standard real-time reverse transcription PCR for SARS-CoV-2.

**Materials and methods.** This retrospective study was conducted at a tertiary care centre from 25 March 2020 to 8 December 2020. The nasopharyngeal/oropharyngeal swabs that were sent to the Viral Research and Diagnostic Laboratory for testing of SARS-CoV-2 by GeneXpert [cartridge-based nucleic acid amplification test (CBNAAT)] were included for the study. A total of 270 samples (220 samples positive and 50 samples negative for SARS-CoV-2 by GeneXpert) were simultaneously tested for real-time reverse transcription PCR. Real-time reverse transcription PCR was considered as gold standard test (reference) for calculating the sensitivity and specificity of the GeneXpert (CBNAAT) test.

**Results.** Out of the total samples tested (*n*=270) for SARS-CoV-2, 220 were positive, and 50 were negative for SARS-CoV-2 by GeneXpert. Among 220 GeneXpert SARS-CoV-2-positive samples, 118 (53.64%) were also positive by real-time reverse transcription PCR, while 102 (46.36%) showed negative results by real-time reverse transcription PCR. However, 50 GeneXpert negative samples showed 100% agreement with real-time reverse transcription PCR, i.e. they were also negative by real-time reverse transcription PCR. The GeneXpert sensitivity and specificity for COVID-19 were seen to be 100% (95% CI: 96.92–100%) and 32.89% (95% CI: 25.50–40.97%), respectively, as compared to the gold standard real-time reverse transcription PCR test. The positive predictive value and negative predictive value of GeneXpert for COVID-19 were found to be 53.64% and 100%, respectively.

**Conclusion.** This study highlights that the GeneXpert test is highly sensitive and found to be useful in emergency and challenging situations for rapidly ruling out negative cases. However, due to its relatively low specificity, positive results should be confirmed with real-time reverse transcription PCR. Therefore, it can serve as a valuable tool for patients requiring urgent care by facilitating early diagnosis and management of COVID-19, ultimately contributing to preventing morbidity and mortality.

## Data Summary

 The authors made available all data obtained from all GeneXpert and PCR runs performed during the months analysed in the study in an Excel spreadsheet format.

## Introduction

The diagnosis of severe acute respiratory syndrome coronavirus 2 (SARS-CoV-2) is very important for early management, contact tracing, and epidemiological purposes [1]. In suspected cases of coronavirus disease 2019 (COVID-19) infection, real-time reverse transcription PCR assay has been validated as the most sensitive and reliable diagnostic assay. The GeneXpert system operates within a closed apparatus, which helps minimize contamination; however, differences in assay parameters, such as Cycle threshold (Ct) cut-offs and target gene detection, may still influence the rate of false positivity [2].

It takes ~24–48 h for the real-time reverse transcription PCR test result as turnaround time, which needs to be done by experienced staff in a containment level 2 (CL2) laboratory set up. With the development of rapid NAAT (nucleic acid amplification test), there has been rapid diagnosis of COVID-19 infection [3]. Under emergencies only (emergency use authorization), the Food and Drug Administration has approved the use of GeneXpert/CBNAAT (cartridge-based nucleic acid amplification test) as recommended by the Indian Council of Medical Research (ICMR) (New Delhi, India) for diagnosis of SARS-CoV-2 (Cepheid Xpert Xpress SARS-CoV-2). Under CL2 conditions and precautions, the test of CBNAAT should be performed, which detects the *E* gene and *N2* region of the *N* gene, which is virus-specific [3]. Both GeneXpert and real-time reverse transcription PCR are based on the same quantitative PCR (qPCR) principle; however, GeneXpert provides results within 1 h, requires minimal resources and can be operated with little to no skilled personnel, whereas real-time reverse transcription PCR requires manual RNA extraction, highly trained staff and up to 2 days to yield results. Additionally, GeneXpert offers a significant advantage in scalability and throughput, making it more suitable for handling large volumes of samples in high-demand situations, such as outbreaks or resource-limited settings.

The recent use of point-of-care tests (POCTs) is helpful in the diagnosis of SARS-CoV-2, as they are molecular real-time quantitative reverse transcriptase PCR assays which are used only for research purposes until the full validation is done, when they can be used for clinical utility in patients as per recommendations from the World Health Organization (WHO). These POCTs include Cepheid’s point-of-care COVID-19 diagnostics, Xpert Xpress SARS-CoV-2 and LAMP test. WHO-approved POCTs have been valuable in monitoring various cases of the pandemic [4,6].

There have been various COVID-19 testing laboratories that have been set up. Approximately 669 laboratories (466 government and 203 private) have been set up in India for COVID-19 testing until 30 May 2020. Of these, 480 labs are using real-time reverse transcription PCR-based tests, and 55 labs are using CBNAAT-based COVID-19 tests [1].

### Aim

The study aimed to compare the performance of GeneXpert against the gold standard real-time reverse transcription PCR for SARS-CoV-2.

## Methods

### Study design

This retrospective study was conducted at Viral Research and Diagnostic Laboratory, Department of Microbiology, Lady Hardinge Medical College and Associated Hospitals, New Delhi, India, from 25 March 2020 to 8 December 2020, for the comparison of GeneXpert to real-time reverse transcription PCR, keeping the latter as the gold standard method. The samples (*n*=220) that gave a positive result by GeneXpert were simultaneously tested for real-time reverse transcription PCR. The 50 negative samples by GeneXpert were also tested for real-time reverse transcription PCR as negative control samples. According to the manufacturer’s instructions, the cut-off value for GeneXpert is 45, whereas according to ICMR, the cut-off value for real-time reverse transcription PCR to be taken is 35. The clinical correlation, like fever, cough, sputum production, sore throat, peripheral oxygen saturation ≤93%, headache and general weakness, is also done along with the testing.

### Sample size

The total sample size is 270 patients with suspected COVID-19 infection during the time period of the second wave of COVID-19 infection.

### Sample processing

The nasopharyngeal and oropharyngeal swabs were obtained from COVID-19 suspected patients by standard techniques and put in viral transport medium (VTM). The swabs were transported in and processed for GeneXpert. After the positivity being obtained by GeneXpert, the 220 positive and 50 negative samples were tested for the gold standard real-time reverse transcription PCR test, and the results were compared. The samples have been stored at −80 °C in a deep freezer. All analysed data were compiled in an Excel spreadsheet format (Table S1, available in the online Supplementary Material).

### GeneXpert procedure

The nasopharyngeal and oropharyngeal swabs are mixed five times by the fast inversion of VTM placed into a transport tube containing 3 ml of viral transport medium or 3 ml of saline. In the sample chamber of the Xpert Xpress SARS-CoV-2 cartridge, the sample was transferred by the transfer pipette. The viral RNA was detected by real-time reverse transcription PCR through automated sample processing after the loading of the GeneXpert cartridge in the GeneXpert Instrument System platform. The results were reported in 1 h after its interpretation by automatic display on the GeneXpert System as positive, negative, inconclusive or invalid. The nucleocapsid gene (*N*) and envelope gene (*E*) were detected by the test. The cut-off cycle threshold (Cq) value was taken as 45 as per the manufacturer’s protocol. Nucleocapsid 2 (*N2*) and envelope (*E*) genes are two targets of SARS-CoV-2 amplified by the Cepheid Xpert Xpress SARS-CoV-2 cartridge. When Cq of both the genes (*N2*+/*E*+) or only *N2* (*N2*+/*E*-) comes during the result, then the test is considered positive, which otherwise is considered as negative (*N2*-/*E*-). Moreover, the *E* gene is only positive in result (*N2*-/*E*+) signifies the *presumptive* positive test when it is necessary for repeat testing. If there is a presumptive positive result on repeat testing (*N2*-/*E*+), according to the manufacturer’s instruction, the confirmatory test can be performed [7]. 

### Real-time reverse transcription PCR procedure

The extraction of RNA was done from the VTM tube (contains Hanks Balanced Salt Solution, heat-inactivated FBS or serum protein components such as bovine albumin fraction and antibiotics) samples as per the manufacturer’s protocol, which was processed through the ‘RNA extraction QIAmp viral RNA Mini Kit’ (QIAGEN India Pvt. Ltd.).

As per the manufacturer’s protocol, the real-time reverse transcription PCR was done by the ‘ICMR NIV Multiplex Single Tube Real-Time PCR Version 3’ kit. The real-time PCR kit includes a set of TaqMan™ reverse transcription PCR assays for the qualitative detection, along with characterization of SARS-CoV-2 RNA. The contents of the kit include the target of SARS-CoV-2 genes (two in number – *E* gene for screening and ORF gene for confirmation) and one housekeeping gene, *β-actin* gene. The 7 µl of extracted RNA was used for amplification, in which the reactions were multiplexed. The enzyme, Taq polymerase, having 5′–3′ exonuclease activity, cleaves the probe. The increase in fluorescence of the reporter is due to the separation of both reporter dye and quencher dye. Therefore, the increase in fluorescence is directly proportional to the target amplification during PCR. The results generated were according to Cq values, as per the standard operating procedures (SOP) by the ICMR ‘Detection of 2019 novel coronavirus (2019-nCoV) in suspected human cases by Multiplex Single Tube Real-Time PCR Version 3’. The results were interpreted as positive (≤35), negative (>35), inconclusive or invalid. The cut-off Cq value was taken as 35 as per SOP by ICMR. The criteria for the interpretation of results have been mentioned in Table 1 as per the ICMR SOP.

**Table 1.** Criteria of real-time reverse transcription PCR result interpretation for SARS-CoV-2 according to genes

Housekeeping gene	SARS-CoV-2 gene		Result
*β-Actin* gene (≤35)	*E* gene (≤35)	ORF gene (≤35)	
Positive	Positive	Positive	Positive
Negative	Positive	Positive	Positive
Positive	Negative	Negative	Negative
Positive	Positive	Negative	Inconclusive
Positive	Negative	Positive	Inconclusive
Negative	Positive	Negative	Invalid
Negative	Negative	Positive	Invalid
Negative	Negative	Negative	Invalid

### Statistical analysis

SPSS Windows version 25.0 software was used for data analysis. A test of significance, like the chi-square test, was applied to find out the results. Real-time reverse transcription PCR is considered the gold standard for calculating the statistical parameters (viz. sensitivity, specificity, positive predictive value and negative predictive value). A value of *P*<0.05 was statistically significant.

## Results

**Fig. 1.**
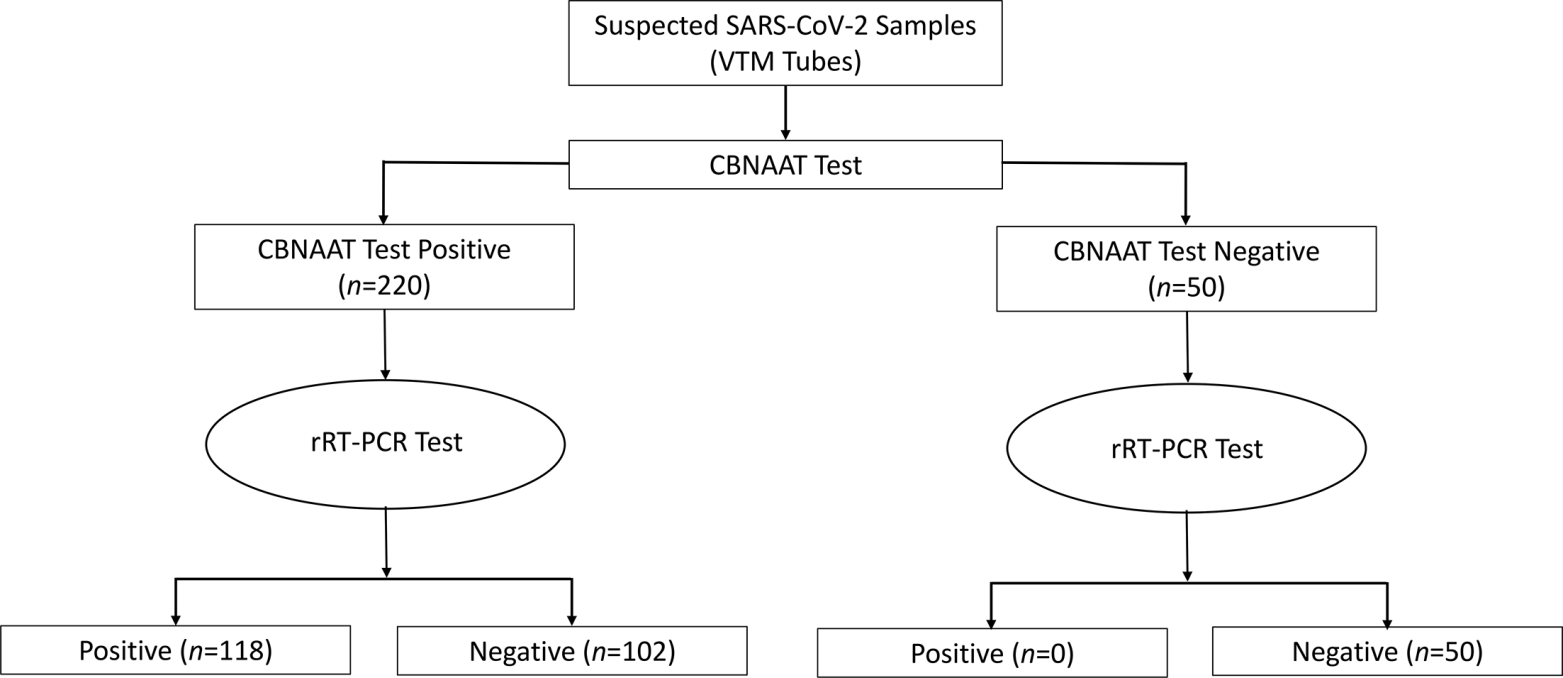
Schematic diagram of the study (*n*=270). rRT-PCR= real-time reverse transcription polymerase chain reaction.

Out of the total samples tested (*n*=270) for SARS-CoV-2, real-time reverse transcription PCR detected 118 positive cases. Among these, all were also positive by GeneXpert. However, GeneXpert additionally identified 102 cases as positive that were negative by real-time reverse transcription PCR. All 50 GeneXpert negative samples showed 100% agreement with real-time reverse transcription PCR, i.e. they were also negative by real-time reverse transcription PCR (Table 2). Fig. 1 presents a schematic diagram explaining the workflow of the study.

**Table 2.** Statistical parameters (sensitivity and specificity) of the GeneXpert test for SARS-CoV-2 detection (*N*=270)

Test	Real-time reverse transcription PCR positive (*N*=118)	Real-time reverse transcription-PCR negative (*N*=152)	Sensitivity (95 % CI)	Specificity (95 % CI)	PPV (95 % CI)	NPV (95 % CI)
GeneXpert positive (*N*=220)	118	102	100% (96.92–100.00%)	32.89% (25.50–40.97%)	53.64% (50.86–56.39%)	100% (92.89–100.00%)
GeneXpert negative (*N*=50)	00	50				

The GeneXpert sensitivity and specificity for COVID-19 were seen to be 100% (95% CI: 96.92–100%) and 32.89% (95% CI: 25.50–40.97%), respectively, as compared to the gold standard real-time reverse transcription PCR test. The PPV and NPV of GeneXpert for COVID-19 were found to be 53.64 and 100% respectively (Table 2).

**Fig. 2.**
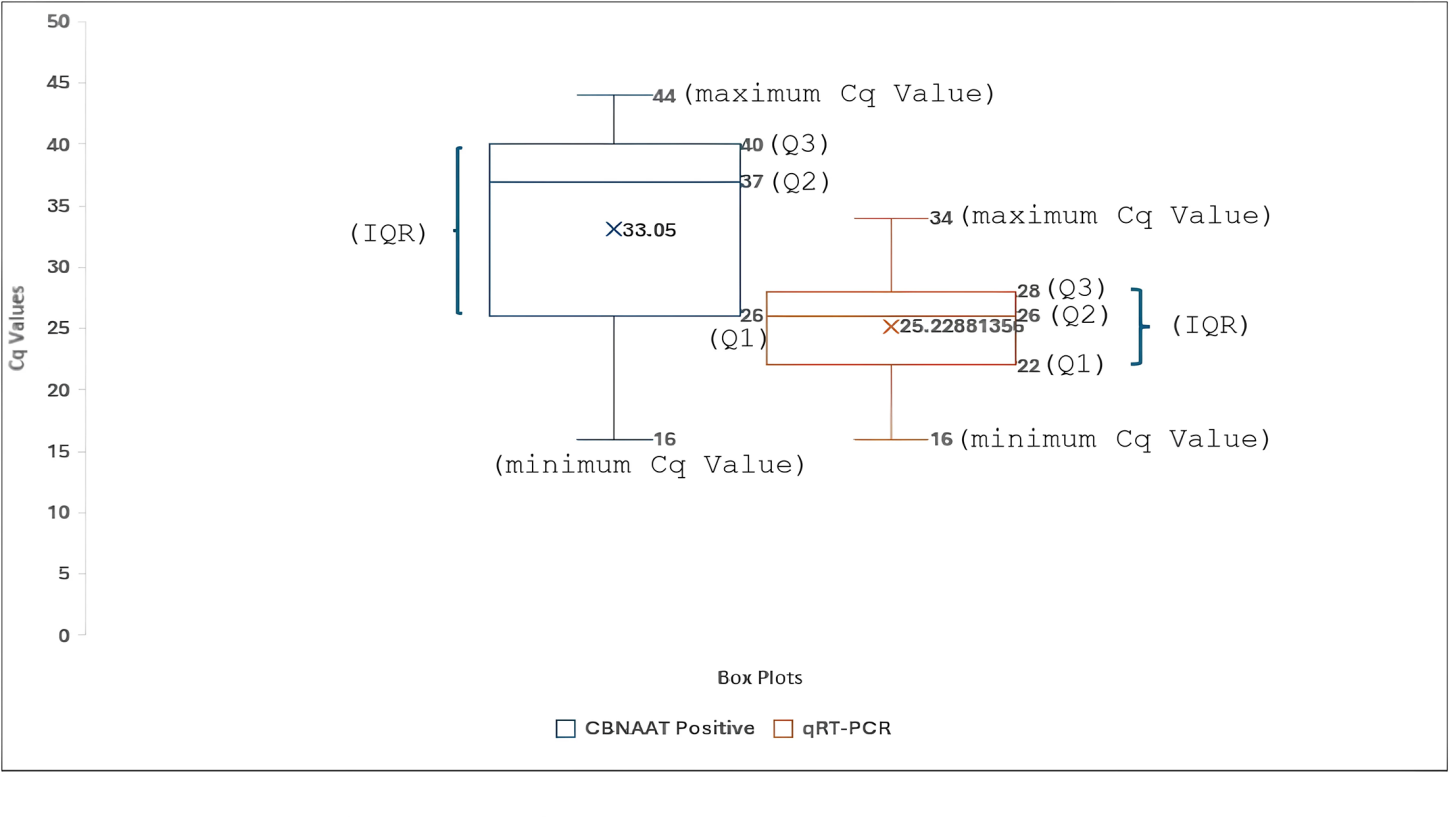
Comparison of Cq values of the GeneXpert positive samples vs. real-time reverse transcription PCR results. The X (cross) in each box represents the mean value, and the central line marking (Q2) represents the median value. Q1, first quartile; Q2, second quartile; Q3, third quartile; IQR, interquartile range (Q3–Q1).

Among the 220 samples positive for COVID-19 by GeneXpert, 102 samples were found to be negative by real-time reverse transcription PCR. It was also observed that all GeneXpert negative cases correlated well with the results of real-time reverse transcription PCR. The comparison of Cq values of the GeneXpert positive with real-time reverse transcription PCR results is shown in box plot graph (Fig. 2). The median Cq values of the GeneXpert (CBNAAT) and real-time reverse transcription PCR positive for COVID-19 are 37 (mean, 33.05; range, 16–44) and 26 (mean, 25.22; range, 16–34), respectively (Fig. 2).

## Discussion

The results presented here suggest that the use of GeneXpert is extremely helpful to exclude disease, as shown by the sensitivity and NPV of 100%. The positive COVID-19 infection shows a positive correlation between the GeneXpert and real-time reverse transcription PCR in patients with Cq values of ≤35, as seen in Fig. 2. Moreover, greater false positivity or less positive predictive value has been noticed with GeneXpert, which interprets the result automatically [8]. Similar results have been noticed in this study, where the positive predictive value is 53.6% (Table 2). Even though there is a difference in the procedure and the primer probes, there is a higher number of positives in GeneXpert. This is due to the variation in cut-offs for positivity and hence the infection rate [9]. The mean and median Cq values of the GeneXpert (CBNAAT)-positive samples are higher than the mean and median Cq values of the real-time reverse transcription PCR-positive samples for COVID-19 (Fig. 2). A similar result was also observed by Sikhosana *et al*. in a study done in 2024 [10]. There are many GeneXpert-positive samples that show high N2 values but are negative for the E gene. Such findings are often observed in samples that test negative by rRT-PCR [11].

As per the results of this study, it is essential to confirm GeneXpert results using real-time reverse transcription PCR. The clinical correlation with a positive GeneXpert result had also been done. The clinical correlation and testing by an alternate method can rule out the false positivity. There are many GeneXpert-positive samples that show high* N2* values but are negative for the E gene. Such findings are often observed in samples that test negative by rRT-PCR [11]. It is still a matter of debate when results are compared with real-time reverse transcription PCR, even though these are different assays with different targets, or the same target but different primers and probes [11]. In this study, there are 46.4% (102/220) cases which are positive by GeneXpert and negative by real-time reverse transcription PCR. These cases had high *N2* and a negative *E* gene. In a study done in Korea, there has been a similarity in the results with 55% GeneXpert positive samples (positive *N2* gene and negative *E* gene), which are negative by real-time reverse transcription PCR [12].

As there is no difference in the degree of infectivity and variation in Cq values, the infection rate is considered with the report of positive results [13,14]. The identification of variance (95 % CI) is an important statistical tool in documenting assay precision. In this study, GeneXpert has shown an overall high sensitivity of 100% (95% CI: 96.92–100%). Rakotosamimanana N. *et al*. [8] in a study done in 2020 stated that the indistinguishable results among the samples, which were positive by both the assays, observed that Cq values were similar in all the samples. The analytical sensitivity is almost similar between both these molecular tests on a broader range, except for a few false negative cases [8]. Since these are commercial assays, the suppliers do not reveal the information about primer/probe sequences.

The detection of SARS-CoV-2 in the initial days of infectivity is essential. It determines the accountability of decision-making in taking quarantine measures for patients who are highly infected at this stage in comparison to infected patients after 10 days of positivity [15]. In our study, the 50 negative GeneXpert samples were also negative by real-time reverse transcription PCR (no false negatives). In a review study done in 2022, the false negative result by real-time reverse transcription PCR is seen to be 0.12 (95% CI: 0.10 to 0.14) [16]. The false negative results are usually due to the improper extraction procedure, reagent or equipment malfunction and the presence of PCR inhibitors in the clinical sample [17]. The other possible reasons can be due to inadequate sample transportation and storage/handling of specimens [18]. The negative test does not rule out the COVID-19 infection. The clinical symptoms and treatment of the patient should be taken into consideration along with the molecular test. The test result also depends on the method of specimen collection like broncho-alveolar lavage, sputum, nasal swabs or throat swabs [18,19].

There is a similar sensitivity of real-time reverse transcription PCR to other tests like computer tomography chest. However, the specificity is on a lower level since similar findings like ground glass opacity are seen in other diseases of the respiratory system [20]. Therefore, it is only recommended for certain medical conditions as the diagnostic test like pulmonary embolism, pneumothorax and other infections of the respiratory system [21]. The point-of-care testing by various serological assays has also shown positive results after about 10 days of symptoms. The test is negative in the early days of infection as antibodies are not developed in the window period [22]. Due to variation in the results of serological tests, these are not used for routine diagnostic tests of acute cases [23]. The Xpert Xpress SARS-CoV-2 test cartridge detects COVID-19 infection in 1 h, which is based on reverse transcription qPCR technique and usually tested in those laboratories that are Clinical Laboratory Improvement Amendments-approvedor patient care set-ups [24,25].

### Limitations of the study

The limitation of this study is the relatively small number of positive GeneXpert samples among the participants. The performance of this assay warrants further evaluation in future research studies. Additionally, the storage of samples prior to testing may have contributed to discrepancies in the results.

## Conclusion

This retrospective study demonstrated the higher sensitivity and lower specificity of the GeneXpert assay for SARS-CoV-2 detection. The higher false-positive rate observed with GeneXpert suggests the need for careful interpretation of results. The discrepancies highlight the importance of further evaluation of upcoming research studies to standardize thresholds and accurately distinguish true positives from false positives, especially in settings using different testing kits. Ensuring uniform interpretation across laboratories is crucial for reliable COVID-19 diagnosis and clinical decision-making. Hence, we recommend that clinicians correlate GeneXpert positive results clinically and consider retesting by real-time reverse transcription PCR over time for confirmation. Additionally, more studies and optimization of GeneXpert thresholds for SARS-CoV-2 detection will be necessary.

## Supplementary material

10.1099/acmi.0.000643.v5 Supplementary Material 1.

## Abbreviations

CBNAAT: cartridge-based nucleic acid amplification test
CL2: containment level 2
COVID-19: coronavirus disease 2019
Cq: cycle threshold
*E*: envelope gene
ICMR: Indian Council of Medical Research
*N2*: nucleocapsid 2 gene
NPV: negative predictive value
POCTs: point-of-care tests
PPV: positive predictive value
qPCR: quantitative PCR
rRT-PCR: real-time reverse transcription polymerase chain reaction
severe acute respiratory syndrome coronavirus 2: SARS-CoV-2
SOP: standard operating procedures
VTM: viral transport medium
WHO: World Health Organization
